# Design of Organic Electronic Materials With a Goal-Directed Generative Model Powered by Deep Neural Networks and High-Throughput Molecular Simulations

**DOI:** 10.3389/fchem.2021.800370

**Published:** 2022-01-17

**Authors:** H. Shaun Kwak, Yuling An, David J. Giesen, Thomas F. Hughes, Christopher T. Brown, Karl Leswing, Hadi Abroshan, Mathew D. Halls

**Affiliations:** ^1^ Schrödinger, Inc., Portland, OR, United States; ^2^ Schrödinger, Inc., New York, NY, United States; ^3^ Schrödinger, Inc., San Diego, CA, United States

**Keywords:** OLED, recurrent neural network, generative model, reinforcement learning, hole transport, organic electronics, molecular simulation

## Abstract

In recent years, generative machine learning approaches have attracted significant attention as an enabling approach for designing novel molecular materials with minimal design bias and thereby realizing more directed design for a specific materials property space. Further, data-driven approaches have emerged as a new tool to accelerate the development of novel organic electronic materials for organic light-emitting diode (OLED) applications. We demonstrate and validate a goal-directed generative machine learning framework based on a recurrent neural network (RNN) deep reinforcement learning approach for the design of hole transporting OLED materials. These large-scale molecular simulations also demonstrate a rapid, cost-effective method to identify new materials in OLEDs while also enabling expansion into many other verticals such as catalyst design, aerospace, life science, and petrochemicals.

## Introduction

We are in a paradigm-changing era in the way scientists develop new materials. The advent of modern virtual screening techniques has benefited from virtually infinite cloud computing resources and then, when combined with modern machine learning techniques, and has demonstrated extraordinary success (Gómez-Bombarelli et al., 2016). In recent years it has become recognized that one could rely on computational chemistry to deliver sufficient accuracy to inform industrial materials research (Yarnell et al., 2021). This new paradigm directly results from the latest advances in the theory and methods of atomic-scale chemical simulation and the expanding computational power described previously.

With the emergence of simulation as a technology driver, improved algorithms for artificial intelligence (machine learning), cloud compute resources, and computational capabilities such as GPUs, TPUs, analog, and purpose-directed (AI/ML) chips (Shalf, 2020) are advancing the utility of simulation capabilities. As a result of these changes, many organizations are expanding their digital transformation efforts (Cheng et al., 2021) to include more efficient data-driven solutions aimed at a significant speed-up in assessing key control variables for the design and commercialization of novel materials (Himanen et al., 2019). With physics-based simulation tools as the foundation, data science helps researchers minimize costly experimental measurements, and computationally intensive simulations (Meredig et al., 2014; Butler et al., 2018) while enabling the utilization of datasets for a more thorough exploration of a materials space (Raccuglia et al., 2016; Ghiringhelli et al., 2017). With these advantages, there is increased use and need for machine learning methods in conjunction with physics-based simulation for various applications in materials research (Pilania et al., 2013; Bartók et al., 2017; Liu et al., 2017; Wei et al., 2019; Moosavi et al., 2020; Nisbet et al., 2020; Xie et al., 2020).

Despite this great potential, there remain challenges such as data scarcity, quality, and the inherent complexity of the model-building and validation procedures that pose a significant obstacle in a data-driven discovery framework. These obstacles often pose a threat to the effective use of the cheminformatics approaches in chemical discovery. Recently software packages have automated best practices for creating predictive machine learning models over chemical data, applying regularization, and searching over appropriate model architectures (Dixon et al., 2016). These packages have successfully provided life sciences applications’ value (Abd El-Karim et al., 2018; de Oliveira and Katekawa, 2018; Murahari et al., 2019; Iwaloye et al., 2020).

The ever-increasing demand for high-performance display technology in consumer electronics drives the design and synthesis of various novel OLED materials, such as charge transporters, hosts, and emitters (Melnyk and Pai, 1990; Jhulki and Moorthy, 2018). Optoelectronic properties, such as HOMO, LUMO, hole reorganization energy, and thermal properties, such as glass transition temperature, are key considerations when designing high-performance OLED materials (Buckley, 2013). Although scientists and engineers have accumulated significant knowledge in synthesizing these materials in the lab over the years, the process of experimentally making and measuring the synthesized structures still requires a considerable amount of effort and time.

Atomic-scale simulation has been an essential tool for navigating the enormous chemical space of novel OLED materials (Halls et al., 2015; Halls et al., 2016). With increasing high-throughput computational capabilities, massive theoretical screening of millions of compounds has become a reality (Matsuzawa et al., 2020a). The ready availability of high-quality computational data generated from simulations is proving to be a gold mine for data-driven prediction of material properties, thereby realizing a significant speed-up in assessing key control variables for designing novel materials.

Although there are several ways to generate ideas for new compounds *in silico*, such as exhaustive R-group enumeration, core hopping, and early structure-based *de novo* design algorithms (Halls et al., 2015), they share many common limitations. One of the most prominent is that generated compounds often do not have the desired properties, including activity, operational stability, and many others depending on the application area despite the intended optimization within the chemical design space. Recent successes with recurrent neural networks (RNN) on SMILES representations of molecules generating predicted activities against dopamine receptor Type 2 (Schneider and Fechner, 2005) have pointed to a new path for molecular *de novo* design targeting specific properties.

In this work, we report a new materials discovery framework powered by the combination of high-throughput quantum chemical simulations and RNN-based generative machine learning techniques designed to explore the chemical space of hole-transporting materials for optimal properties. Successful demonstration of a goal-directed machine learning approach for the case study is realized by uncovering novel materials chemistry satisfying the design criteria.

## Methods and Principles

### Goal-Directed Generative Machine Learning

We built a goal-directed generative model using the REINVENT (Olivecrona et al., 2017) protocol, which has shown success in drug discovery applications (Ghanakota et al., 2020) by generating tens of thousands of unique structures with targeted properties while only requiring a few hours of computing time. The deep reinforcement learning methodology applied in the REINVENT protocol is a robust solution for chemical enumeration while not consuming a prohibitive amount of computation cost (Olivecrona et al., 2017; Ghanakota et al., 2020).

**FIGURE 1:**
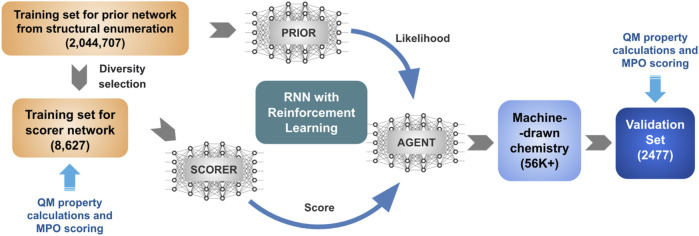
Schematic representation of the goal-directed generative model for materials discovery.

The general schematics of the goal-directed generative model in Figure 1 describes a protocol of two individual stages. The first stage trains a prior network from an extensive collection of structures in the chemical space of interest. The second stage shifts the distribution based on a utility function, encoding the desired property ranges. In previous studies, we trained the REINVENT algorithm with a group of structures generated by the PathFinder algorithm (Ghanakota et al., 2020). This work aims to use a design space for REINVENT to cover organic electronics, represented by the popular structural motifs shared among successful hole transport materials.

Hole transport materials cannot be successfully designed based on a single design parameter. Most material designs require consideration of multiple parameters that may or may not be coupled to each other by a complicated network of trade-offs. We developed a multiparameter optimization (MPO) scoring function to cope with design principles. Four critical hole transport performance parameters were selected and used to construct the property space, namely two molecular orbital energies (HOMO and LUMO) that define the band offset of the hole transport layer, hole reorganization energy that dictates charge carrier (i.e., hole) mobility, and glass transition temperature (*T*_*g*_) that determines morphological stability. The property space was projected to a single MPO score and used as the utility function for the REINVENT algorithm generating new chemical structures described in Figure 1.

### Chemistry by Neural Network

As demonstrated in previous drug discovery work (Olivecrona et al., 2017), the prior network provides a mechanism to create new chemical structures powered by a deep neural network. A training set library defines the chemical design space. Still, it is crucial to recognize that one cannot simply reuse a prior network explicitly used for drug design (e.g., ChEMBL database). An entirely new training set that resembles more of the chemical space for a target material must be used, such as the one needed in this work.

We collected two groups of chemical structures composed of cores, and R-groups, to assemble the common structural motifs of known hole transport materials for building a prior network. R-groups are defined by the chemical substructures observed frequently on the structural periphery of known hole transport materials, and each is given a single attachment as a connection point to a core. The core and R-group setup can provide an extensive chemical library base through simple structural enumeration while preserving the chemical space of designers’ interest. The symmetric enumeration scheme also adds the resulting chemical structures with high symmetry, translating into better operational stability and, to a certain degree, and enhanced synthetic viability for the resulting design space (Voršilák et al., 2020).

**FIGURE 2:**
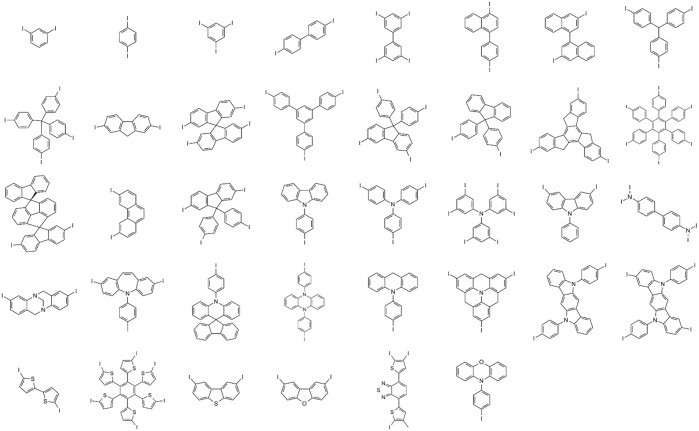
Thirty-eight unique core structures curated from a known group of hole transport materials as the basis of the training set for building a prior network. Attachment points for R-group enumeration are marked with I.

We manually selected thirty-eight unique cores (Figure 2) from frequently appearing fragments in known hole transport materials published in commercial catalogs and literature (Shahnawaz et al., 2019). We enumerated the structural R-groups with a genetic algorithm featured in the Materials Science Suite as a goal-directed chemical design solution (Schrödinger, 2021; Jennings et al., 2019), starting from a group of similarly found fragments reported in the hole transport materials literature. The same genetic optimization tool implemented in this work has already found utility for designing new molecular materials for battery technology (Murdock et al., 2015). We used the genetic algorithm as a library generation tool without explicit goals to generate 53,808 unique R-groups defined in the organic electronics space. The unique R-group set used in this work to build the training set for the prior network is available from files provided with the supporting information.

The Materials Science Suite (Schrödinger, 2021) enumerated R-groups based on the core and R-group libraries. The structural enumeration steps provide symmetrically equivalent groups of substitution points that will always get the same R-group, while each of the R-groups can be substituted independently. Based on the principles of structural enumeration, over 2 million structures were generated and used for training the prior network.

### High-Throughput Quantum Chemistry

Once a prior network defines the chemical design space, the next step is to build a training set for the scorer network of the generative model. The role of the scorer network is to provide the context of structure-property relationships to the model, so the machine can accurately differentiate useful chemistry from less-useful chemistry. Training the scorer network requires a property space paired with the chemical space. This building process is similar to a conventional quantitative structure-property relationships (QSPR) model. It is difficult to find or generate a large amount of reliable data, and consequently, this data scarcity is a challenge when building an accurate machine learning model.

One of the emerging trends in overcoming the data scarcity problem in chemical machine learning models is to utilize physics-based theoretical predictions of the material’s properties as a virtual training set. These simulations are now possible because of recent improvements in predictive accuracy and that one can cost-effectively access such predictions at a large scale. A recent example of organic semiconductor design (Matsuzawa et al., 2020b) demonstrates how the state-of-the-art physics-based simulation technology powered by a large-scale computing environment accessible to the scientific community can be utilized to produce a large number of reliable materials databases for the development of new materials. The automated large-scale quantum chemical and machine learning predictive schemes used in work are designed to utilize a similar strategy in developing new organic electronic materials.

Using a diversity selection method (An et al., 2012) featured in Schrödinger’s MS Informatics package, 265 representative structures were selected from each of the 38 cores, resulting in a total of 10,070 structures. We ran geometry optimization of all 10,070 structures with the same parameters to calculate orbital and reorganization energies. Any compounds that have failed to produce a converged low-energy geometry were removed from the list, leaving 8,627 compounds to be selected and used as the training set for the scorer model. The Materials Science Suite optoelectronics workflow module was used to compute orbital energies (for HOMO and LUMO) and hole reorganization energies of the training set compounds. The raw orbital energies computed from density functional calculations are rarely comparable to the ones obtained from the experiment. This is because the experimental orbital energies are often derived from measured redox potentials using the following expressions to assess the difference between the electrode energy and the redox potentials:

$$V_{\text{Abs}\text{. Electrode}}{=\text{ E}}_{\text{NHE}}\;+\;V_{\text{Electrode}}$$ (1)

$${\text{E}}_{\text{HOMO/LUMO}}\;=\;V_{\text{Abs}\text{. Electrode}}-V_{\text{Oxidation/Reduction}}$$ (2)

Shukla and coworkers (Shukla et al., 2009) and Kondakova and coworkers (Kondakova et al., 2008) suggested that a relatively inexpensive level of theory and basis set (B3LYP/MIDI!) combined with empirical corrections from molecular density functional calculations can generate the orbital energies (Koopman redox potentials) that are more comparable to the experimental measurements. We apply the empirical corrections to produce the Koopman redox potentials from the raw density functional orbital energies as follows:

$${\text{V}}_{\text{Oxidation}}\;=\;-17\text{.}50\cdot {\text{E}}_{\text{HOMO, DFT}}\left(\text{eV}\right)-2\text{.}17$$ (3)

$$V_{\text{Reduction}}=\;-22\text{.}50\cdot E_{\text{LUMO, DFT}}\left(\text{eV}\right)-\;3\text{.}21$$ (4)

These redox potentials are then entered into Eqs 1, 2 to produce the orbital energy predictions. *E*_NHE_ is the energy of the NHE electrode in water, taken to be −4.28 V (Truhlar et al., 2004; Kondakova et al., 2008), and *V*_Electrode_ is the potential of the chosen electrode relative to NHE. We used the V_Electrode_ of 0.25 V in this work, which is the potential relative to the saturated calomel electrode (SCE). This results in the absolute electrode potential (V_Abs. Electrode_) of −4.53 V. Hole reorganization energies were computed using the following equation:

$${\text{λ }}_{h}=\left(E{\;}_{\;relaxed\;at\;cation}^{neutral}-E{\;}_{\;relaxed\;at\;neutral}^{neutral}\right)+\left(E{\;}_{\;relaxed\;at\;neutral}^{cation}-E{\;}_{\;relaxed\;at\;cation}^{cation}\right)$$ (5)

The reorganization calculations were performed at the same level of theory without further empirical corrections, which provides accurate predictions in the hole transport materials space based on a previous study (Evans et al., 2016).

**FIGURE 3:**
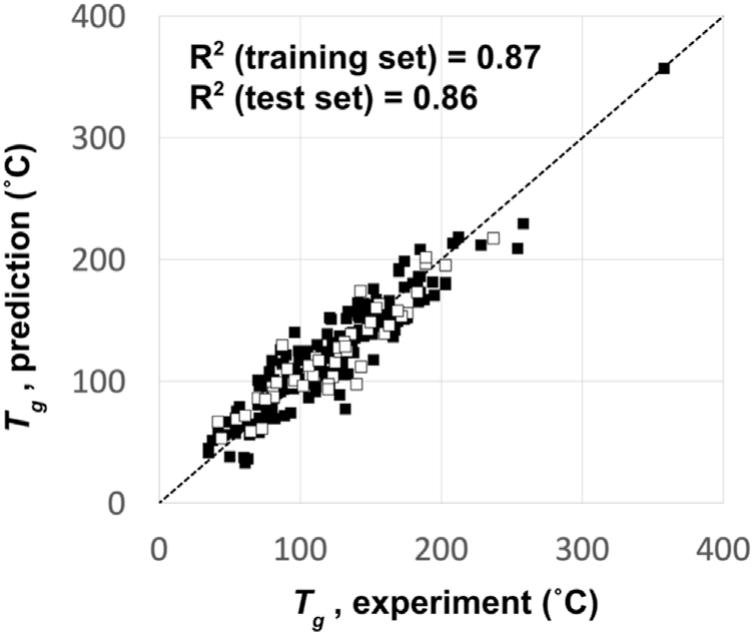
QSPR model predictions vs experiment for the glass transition temperature (T_g_) of the 250 OLED compounds used to build the T_g_ prediction model. Filled squares represent the training set data, and empty squares represent the test set data.

Owing to the computational cost of predicting glass transition temperatures (*T*_*g*_) using physics-based simulations (Afzal et al., 2021), we opted to use a QSPR model instead. The QSPR model was trained to predict *T*_*g*_ based on 250 known OLED materials from the literature (Naito and Miura, 1993; Fujikawa et al., 2000; Shirota, 2000; Yin et al., 2003; Kimura et al., 2005; Xu and Chen, 2005; Gao et al., 2007; Shirota and Kageyama, 2007). A Kernel-based Partial Least Squares (KPLS) method (An et al., 2013) was used to build a predictive model that results in an R^2^ for the training set compounds of 0.87 and that for the test set compounds of 0.86. A training-set to a test-set ratio of 4:1 was used to assess the model’s accuracy to unknown chemistry. Figure 3 shows the comparison between the QSPR predicted and experimentally measured *T*_*g*_ of the 250 OLED compounds used to build the QSPR prediction model.

### Multiparameter Optimization

**FIGURE 4:**
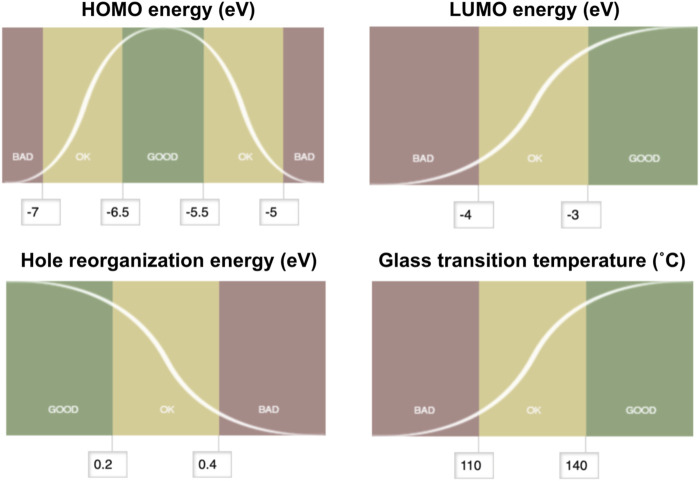
Desirability score setups for the multiparameter optimization scoring of hole transport materials. HOMO energy (upper left) is set up with the “middle good” mode, hole reorganization energy (lower left) is set up with the “lower better” mode, and both LUMO energy (upper right) and glass transition temperature (lower right) are set with the “higher the better” mode.

Multiparameter optimization, often referred to as MPO, provides a measure to assess the logistic transformation function for each constituent property. The MPO scheme used in this work translates each property data to a dimensionless scale between 0 and 1. The MPO scores are set up so that the compound would score below 0.2 if it is bad, between 0.2 and 0.8 if it is ok, and above 0.8 if it is good, and based on the user-specified criteria. The MPO score itself is continuous as defined by the curve for any given set of properties that consist of the multiparameter space. The first step to convert a set of individual properties into a single MPO score is to normalize each individual property into a dimensionless score that ranges from zero to one. We define this normalized score as the desirability score. Conversion from the individual properties to the desirability score is done using a logistic function, whose shape can be further tuned by defining fixed boundaries between bad and good values. In each property space, we define a specific logistic function f(*x*) for the property *x*. This is done by constraining the value of f(*x*) as 0.2 at the boundary between ok and bad values for *x*, and as 0.8 at the boundary between good and ok values for *x*. These points correspond to the inflection points on the logistic function curve, as shown in Figure 4. With these constraints, one can solve the logistic function written as below for a and b for each property space:

$$f\left(x\right)=\frac{1}{1+e^{-b\left(x-a\right)}}$$ (6)

If the property needs to be maximized (identified as a “higher better” mode with b > 0) or to be minimized (identified as a “lower better” mode with b < 0), a single logistic function can be used to set up the desirability score per property space. If the target property is sandwiched between the less-desired property domains (identified as a “middle good” mode), we break down the property space into two spaces and solve for the two logistic functions—“higher better” mode on the left and “lower better” mode on the right—that are facing each other in line symmetry at the target property. Once the logistic function f(*x*) is solved for each property space, f(*x*) becomes the desirability score for a compound that has the property *x*. The MPO score is then generated by taking the geometric mean of the desirability scores.

$${\left(\;\overset{n}{\underset{i=1}{\prod }}a_{i}\right)}^{\frac{1}{n}}=\sqrt[{n}]{a_{1}\cdot a_{2}\cdots a_{n}}$$ (7)

Here, *a*_i_ is an individual desirability score for the *i*-th property of a compound, and *n* is the number of individual properties that get combined to produce a single MPO score, where *n* = 4 in this work. The MPO scoring scheme was integrated as part of the multiparameter optimization solution of LiveDesign (LiveDesign, 2021) for automation. We note that the geometric mean is used in this work to produce MPO scores instead of the arithmetic mean because the arithmetic mean could cause a dramatic shift in the MPO score by just one of the target properties being an outlier. This could be detrimental to the quality of the generative model, as a single property labeled as bad can disqualify the compound regardless of how high it scores on other properties.

Figure 4 summarizes how the desirability scores for each of the four hole transport materials properties examined in this work were set with target values and cutoffs. HOMO energy scores were defined such that a maximum score in the “middle good” mode would be for a target property of −5.5 eV. Deviations from this target value by 0.5 and 1.5 eV were set as the boundaries between good/ok and ok/bad regions, respectively. LUMO energy scores were set with the “higher better” mode to be a maximum value of −3.0 eV set as good while below −4.0 eV was set as bad. Hole reorganization energy scores were set with the “lower better” mode to minimize the value. We observed known hole transport materials with high charge mobilities with a hole reorganization energy less than 0.3 eV (Evans et al., 2016); thus, compounds with reorganization energy below 0.2 eV were defined as good while those above 0.4 eV were defined as bad. The score was set for the glass transition temperature relative to a “higher better” mode that maximizes *T*_*g*_. The upper and lower boundaries between good/ok and ok/bad were set as 140°, 110°C, respectively.

## Design by Generative Model

The prior network from the generative model created more than 20 million chemical structures over 40 h of model generation on a single general-purpose GPU card (NVIDIA GeForce GTX 1080). REINVENT also generated 56,290 unique chemical structures when optimizing for MPO score. Further inspection of the generated structures indicates that 93.2% (52,469) of the structures are predicted to have an MPO score of 0.8 or higher. Such high scores on the chemical structures from the generative model provide direct measures for the model performance. Using the diversity selection algorithm featured in Canvas, 2,477 representative candidates out of the 50 K + structures were further selected for validation. In this work, the group of candidates was used both as the validation set for the MPO score and as the representative design pool for the new hole transport materials. The newly generated chemical compounds from the generative model were predicted with an MPO score higher than 0.8 (56,290). The validation set selected from the diversity selection (2,477) is provided in the supporting information.

### Validation in Property Space

**FIGURE 5:**
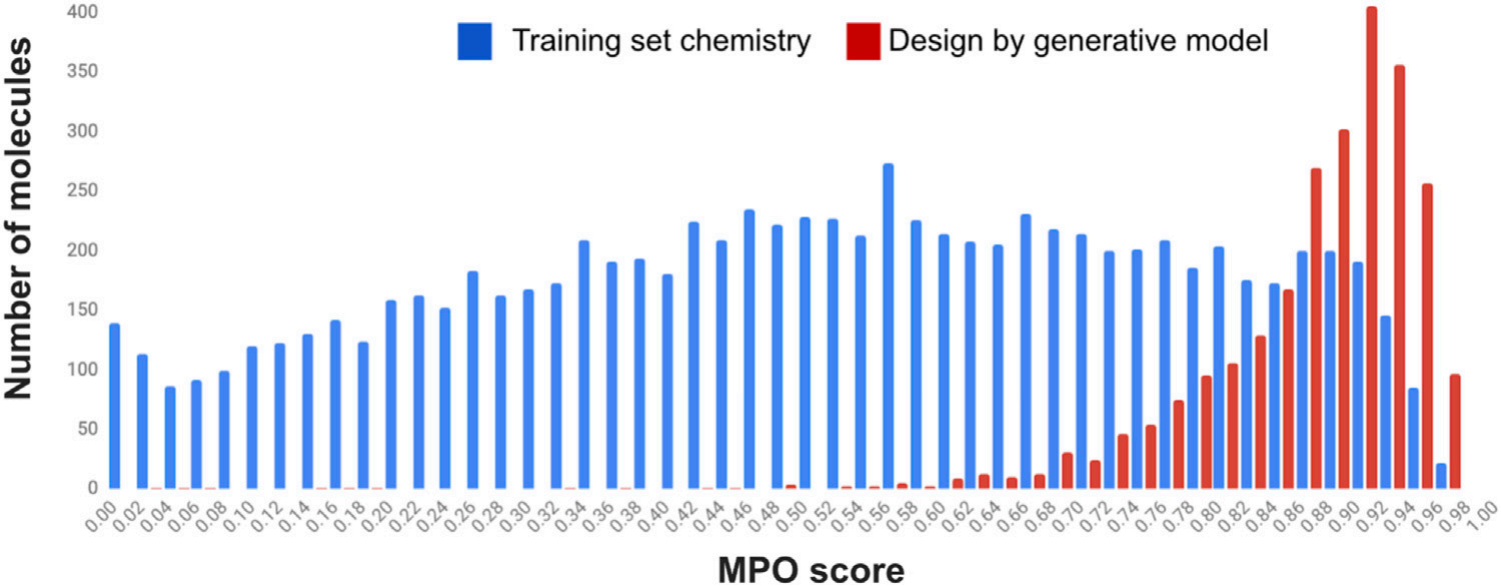
MPO score distribution (histogram) of the training set for the scorer network (blue) and the validation set generated by the generative model (red).

We assessed the accuracy of the structure-property relationships predicted by the generative model’s MPO score for the validation set of 2,477 candidate compounds. MPO scores estimated for the validation set compounds were compared to the properties (i.e., orbital energies and hole reorganization energy by quantum chemical calculations and *T*_*g*_ from QSPR predictions) of the 8,627 training set compounds used to build the scorer network. A comparison of the distributions of MPO scores between the training set for the scorer network and the validation set created by the generative model is shown in Figure 5. It is clear, as shown in the figure, that the agent network from the generative model is fully capable of generating a large number of chemical structures that exceed property scores compared to the chemical structures used as the starting point.

We note the MPO scores shown in the figure for the validation set are not the scores produced by the agent network from the generative model as part of the model predictions. Instead, the MPO scores were individually recomputed based on the properties of each compound through quantum chemical calculations (for the orbital energies and the reorganization energy) and QSPR predictions (*T*_g_). We find that nearly 84% of the remaining validation set compounds (2,088) score greater than or equal to 0.8 on the MPO score after the reevaluation of the individual properties. The MPO score of 0.8 in this work is designed to be the indicator that differentiates a good design from a bad design, and the number of compounds among the validation set with the MPO score greater than 0.8 would provide the predictive measure of the structure-property relationship model used within the generative model itself.

The results presented in the MPO score distribution indicate that a generative model can design new chemistry while significantly pushing the property profile into the desired MPO score range of 0.92 as compared to the property space from the training set where the MPO score is almost evenly distributed between 0 and 1.

**FIGURE 6:**
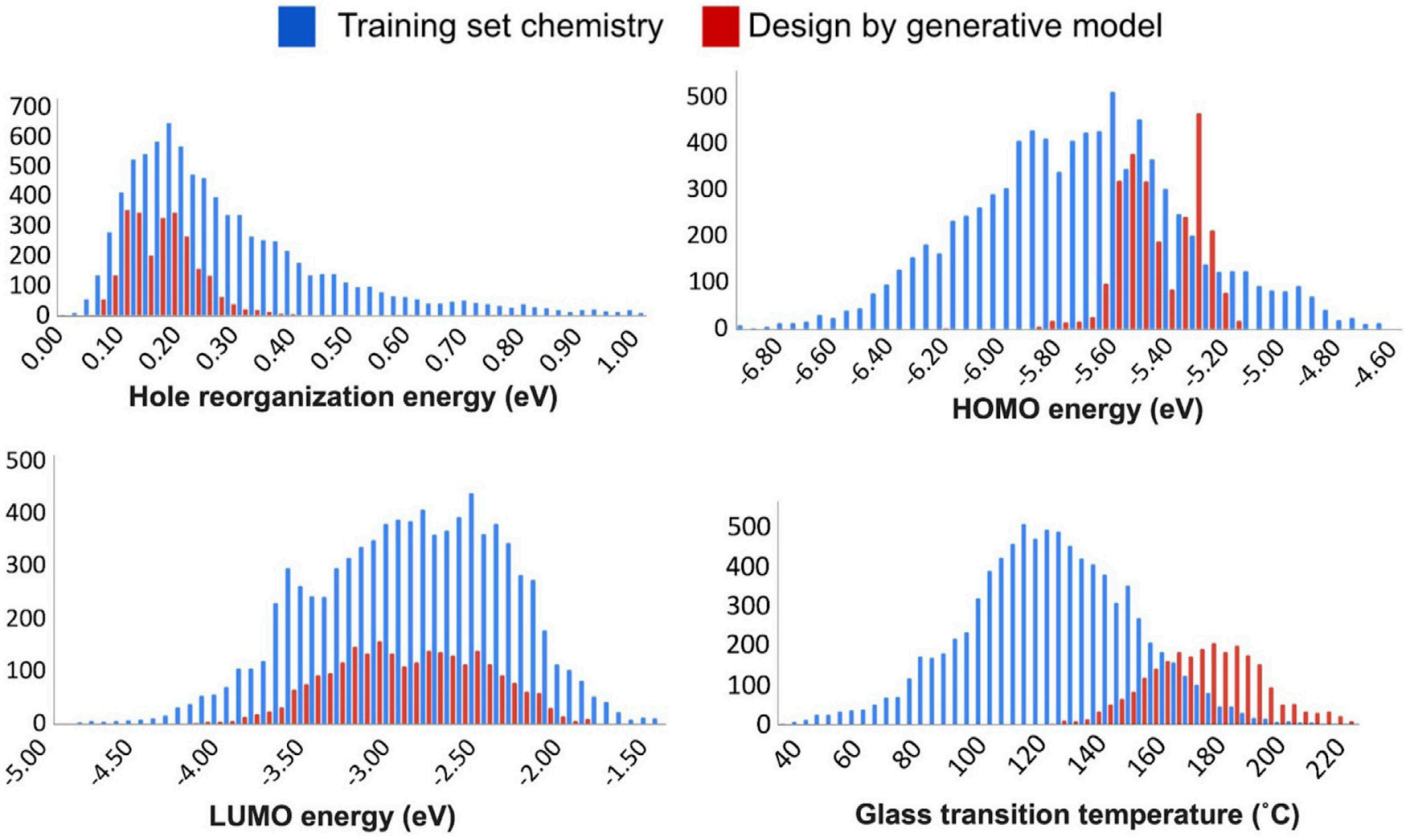
Comparisons between the training set chemistry (blue) and the chemistry created by the generative model (red) over the four individual design properties considered in this work - hole reorganization energy (upper left), HOMO energy (upper right), LUMO energy (lower left), and glass transition temperature (lower right).

We compared the individual design properties between the training set chemistry and the generative model chemistry, see Figure 6. Comparisons of the percentages within the target space between the training set and the validation set chemistry are summarized in Table 1. Overall, we found that individual properties and MPO scores are generally in the expected target property range. Further analysis revealed two different types of variation in the property space derived from the generated chemistry model. The first type is where the newly developed chemistry becomes enriched in a property space corresponding to the more desirable hole reorganization energy and the LUMO energy.

**TABLE 1:** Percentages of chemical compounds from the training set and the validation set generative chemistry model bound within the target space.

Property	MPO	*E*_HOMO_ (eV)	*E*_LUMO_ (eV)	*λ*_*h*_ (eV)	*T*_*g*_ (°C)
Target interval space	[0.8, 1]	[−5.7, −5.3]	[−3.0, 0.0]	[0.0, 0.2]	[140, ∞]
% in training set chemistry	16.1	32.3	59.0	36.9	23.0
% in design by generative model	84.1	86.1	55.4	70.9	95.5

In contrast, a significant reduction in the population of less-desired properties is observed. There is an enrichment in the desired property space marked with an improved distribution corresponding to ideal HOMO energy and glass transition temperatures in this second grouping.

For the hole reorganization energy, most of the validation set chemistry from the generative model shares a hole reorganization energy below 0.2 eV. The training set chemistry was constructed with substructures from known OLED chemistry. Consequently, the hole reorganization energies were already well regulated in the training set space. As such, there was little need to drive down the average value for the reorganization energy for the newly generated chemistry. Instead, it is more desirable to minimize the number of compounds generated with the reorganization energies that are much higher than the mode of the distribution. This is indeed the observed trend from the newly generated chemistry in Figure 6.

Orbital energies show signs of improvement from the generative model by delivering an enriched LUMO energy and favorable shift of the HOMO energy. However, a couple of observations cannot be simply addressed as improvements in the property space. The HOMO energy, for example, of the validation set lies close to the target property, and it shows a bimodal distribution with fewer centered at the desired value of −5.5 eV versus the two modes centered around −5.56 eV and −5.32 eV. In the LUMO case, the generative model does not seem able to push the LUMO energy much higher than the starting point. Instead, it can be seen from the summary in Table 1 that the generative model returns a reduced number of compounds from the target property space.

This performance indicates a specific limitation, and hence, requires a close inspection of the generative model by the user. In this case, a generative model scheme improved molecular electronic property prediction. However, it is essential to note that some property subspaces may be inherently unapproachable by an initially defined and trained prior network. Again, a close inspection by the user of each property is required before assessing the model’s validity. Further, while LUMO energy space did not seem to show as impressive an improvement as other property space, the starting point for LUMO energy already had the highest percentage of structures in the training set (59%) that meet the design criteria. As such, maintaining the equivalent ratio of chemistry that meets the criteria while simultaneously satisfying other critical design criteria, and retaining a high MPO score, was still deemed a significant success.

The glass transition temperature can illustrate the most straightforward improvement among the individual target properties that drive the generative model. The validation chemistry from the generative model pushes the average *T*_*g*_ towards the upper-bound of the starting distribution from the training set while resulting in a significant shift of the average *T*_*g*_ expected from the materials by nearly 60°C, with over 95% of the new materials designed by the model satisfying the property target (*T*_*g*_ > 140°C).

### Validation in Chemical Design Space

One advantage of using recurrent neural networks in combination with reinforcement learning for a generative chemistry model (Olivecrona et al., 2017) is the ability to generate valid chemical structures with a much higher success rate than traditional methods such as variational autoencoder algorithms (Alperstein et al., 2019; Jewell et al., 2021). For example, the convolutional variational autoencoder (CVAE) has been widely known among the drug discovery community as a fast and efficient tool to generate new molecular structures. Yet, the approach is often challenged by the low success rate in generating valid chemical structures, let alone ones with the desired properties (Lim et al., 2018). On the other hand, we confirmed that over 80% of the two million-plus SMILES strings generated by the prior network in this work were valid chemical structures, and rendering the rate of developing valid chemistry a non-issue for this framework. Optimization in the property space has also proven more successful with the RNN based generative design, as reported in benchmark studies (Brown et al., 2019; Marques et al., 2021).

As the first step to understanding the design space, we examined the overall similarity between the chemical structures from the training set for the scorer network and the validation set generated by the model. Similarities between chemical structures were quantified by a Tanimoto distance metric that is a normalized measure of the similarity in descriptor space between two molecules:

$$Similarity=\frac{\sum_{i}x_{i}^{B}x_{i}^{A}}{\sum_{i}x_{i}^{A}x_{i}^{A}+\sum_{i}x_{i}^{B}x_{i}^{B}-\sum_{i}x_{i}^{B}x_{i}^{A}}$$ (8)

where *A* and *B* are indicators for two different molecules in comparison (with *A* being the reference molecule). In this work, we use extended-connectivity fingerprints from RDKit (Rogers and Hahn, 2010) as the descriptor space for estimating the similarities, and as such, *i* denotes an index for the fingerprint bit. The similarity value per pair of molecules lies between one and zero based on the definition. One indicates identical molecules, while zero indicates a complete dissimilarity. The primary purpose of the analysis is to assess the novelty of the design space quantitatively. In an ideal scenario, the candidate materials space created by the generative model would be 1) close enough to the training set space to retain the original design space characteristics (*e.g.*, organic compounds with a resemblance to hole transport materials) and 2) far enough from the training set space to have an improved set of properties as predicted by the machine learning algorithms.

Five hundred top-ranked structures were selected based on the MPO score from the newly generated structures in the generative model. Then, for each of the structures, a new chemical structure with the highest Tanimoto similarity was selected based on its MPO score:

$$\Delta MPO\;=\;MPO_{G}-MPO_{S}$$ (9)

where,

$$MPO_{G}=MPO\left\{newdesigncreatedbythegenerativemodel\right\}$$ (10)

$$MPO_{S}\;=\;MPO\left\{closestsiblingfromthetrainingset\right\}$$ (11)

**FIGURE 7:**
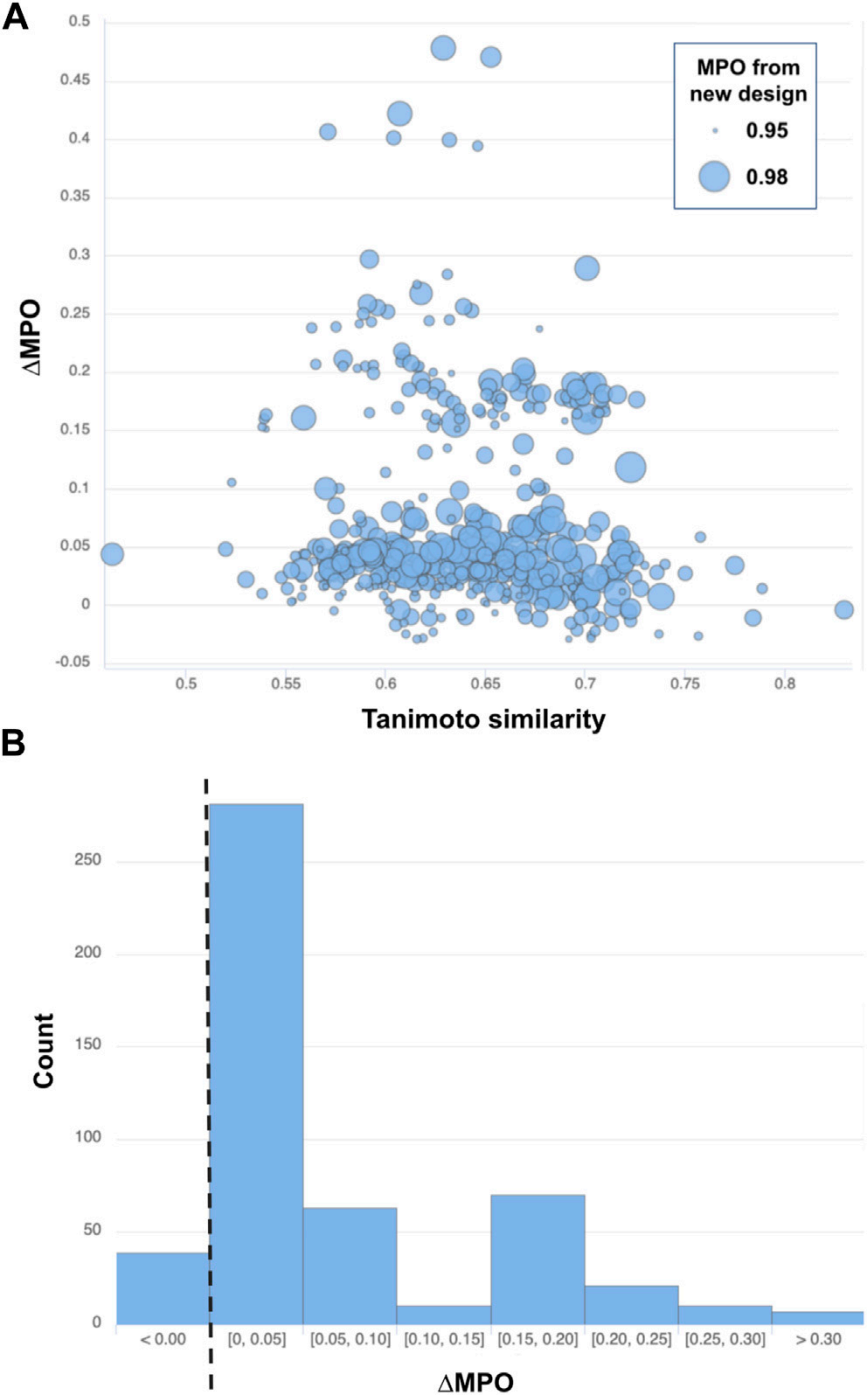
**(A)** Similarity vs ∆MPO of the top-500-ranked new designs by MPO score and **(B)** the distribution of ∆MPO shown as a histogram with a dashed line marking the point of no improvement (i.e., ∆MPO = 0). Each data point from **(A)** is drawn with a circle whose size scales to the MPO score of the newly designed target.

Note that ∆MPO >0 indicates improved properties with the new design but an ∆MPO <0 is a decline in the design properties. Figure 7A summarizes the difference in MPO scores for all 500 pairs of structures picked from the top-ranked validation set compounds and the corresponding (closest-resembling) training set compounds. Figure 7B also shows the distribution of the MPO score difference by a histogram.

Most of the structure pairs shown in the plot mark a similarity score greater than 0.50 on the *x*-axis, confirming a solid resemblance between the newly generated structures and the starting space for those designs. The next thing to notice is that most of the data points (461 out of 500, or 92.2%) shown in Figure 7 lie above the line ∆MPO = 0, implying the improvement of the target properties over the closest representations from the training set space. The maximum ∆MPO is around 0.5, while the minimum ∆MPO score is less than around −0.03. So, the extent of improvement is much greater than decline. Its also noteworthy that the most significant improvement in the property space occurred when the similarity between the closest siblings in training and validation sets ranges from 0.55 to 0.70.

**FIGURE 8:**
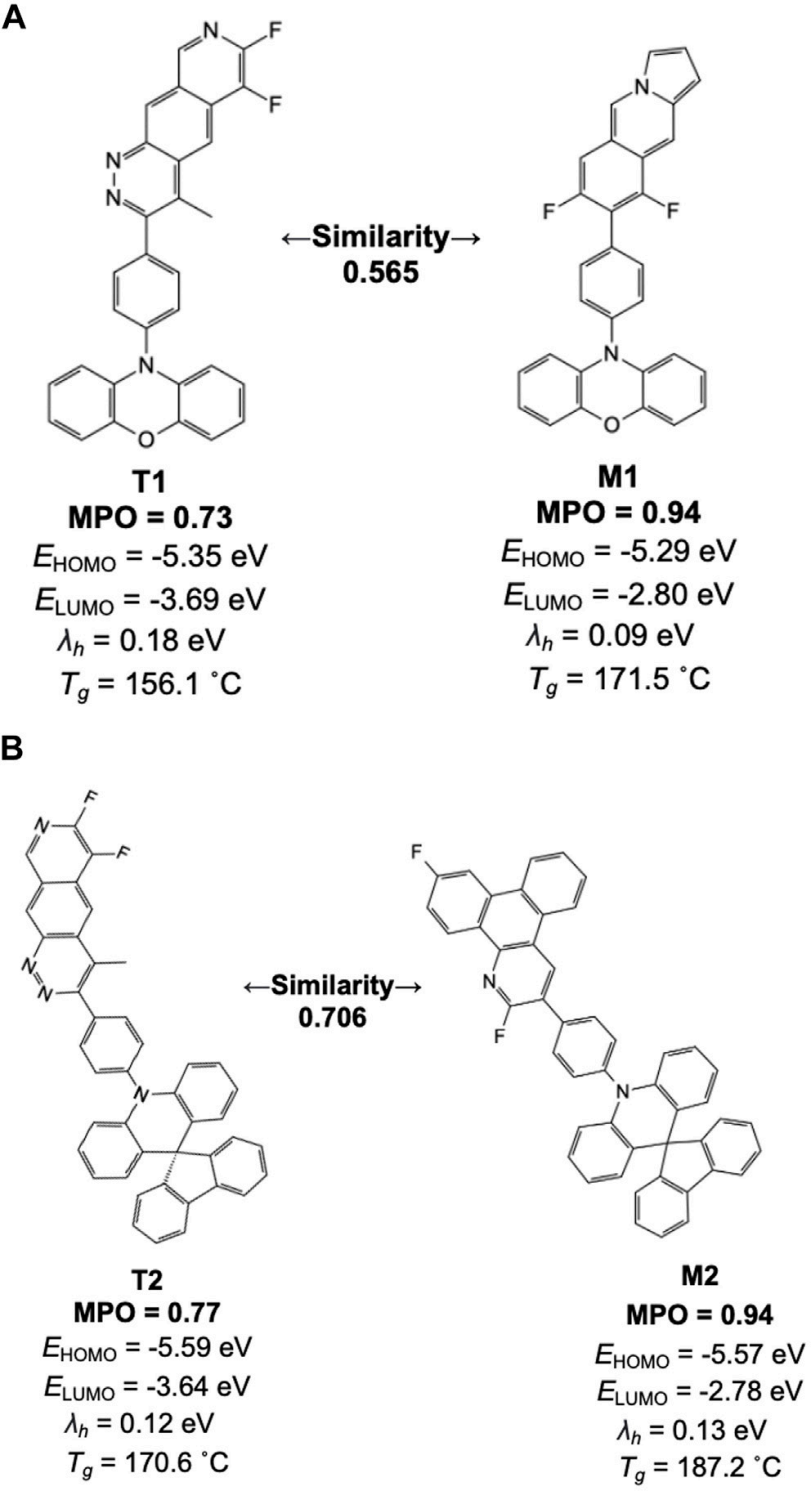
Machine-drawn examples created by the generative model (**M1** and **M2**) and their counterparts in the training set space for the scorer network with the highest similarity (**T1** and **T2**) are shown with individual properties and their MPO scores. **(A)** Similarity 0.565, **(B)** Similarity 0.706.

Figure 8 lists a couple of example pairs selected from the similarity analysis. One derives from the high-scoring machine-generated design, and the other from a training set space where the scorer network resembles the machine-generated design. Inspecting the individual chemical structures generated by the model with high scores, we find many of the structures contain one of the 38 original core chemistries provided as the basis for building the prior network. In Figure 8, for example, two apparent core structures have driven the generative and training set chemistry models into relatively higher MPO ranges than average. This result is consistent with the general observation that the generative molecular structures are defined by core-fragments found in the training chemistry and the generative molecular structure space. This introduces a constraint on structure diversity in this study but will be the subject of future work.

We note a unique aspect of the design results in an increase of MPO score by nearly 0.2 while there is no apparent structural correlation between the training and the validation set compounds. In most of these cases, comparing the training set chemistry and the generative model design points to a trend wherein the structural features not shared by the two are often alien to the 38 core structures used to build the prior network. This often leads to a reduced number of symmetry points and, thus, increases the uniqueness of each design. Based on this aspect, newly generated molecular design by the generative model can benefit from the known chemical design space that is proven to work while suggesting new design ideas to push the target property space even further. More specific design strategies to accomplish this will be discussed in the later section in more detail.

One of the desired traits of the generative model for chemistry is to have the capability to extrapolate target properties beyond the range that the training set data provides. While the MPO scoring scheme we used in this work is not designed explicitly to support the capability of extending the score outside the training set domain—*i.e.*, by confining them with the definition of the MPO score to be between 0 and 1—individual properties are not strictly bound by such constraints and still have the freedom to result in an extrapolation. In fact, we observe in Figure 6 the sign of extrapolation in the glass transition temperature with the newly generated compounds, as the validation set analyzed in this work has 17 compounds with *T*_*g*_ greater than the upper limit from the training set (215°C). This is supported by the previous study where the REINVENT formalism was used to create the new molecular designs (Marques et al., 2021), reporting the model’s general capability of extrapolating outside the training set domain. Based on our observation, we believe the best strategy to bring the extrapolation capability to the generative model is 1) to take advantage of multiparameter optimization, 2) to maximize the number of training set data to provide enough resolution in the property space to extend near the edges of the training set domain, and 3) to have the property prediction model—such as quantum chemistry for electronic properties and QSPR for glass transition used in this work—available well outside the training set space to keep the accuracy of predicted properties for the validation sets high.

### Validation by Chemical Stability

Chemical stability is an essential design for any novel organic electronic material. Bond dissociation energy (BDE) of the weakest bond for a chemical compound is often considered a good predictor of chemical and operational stability. An explicit demonstration for hole transport materials was previously described by Kwon and coworkers and Kondakov and coworkers (Lee et al., 2021). Unfortunately, there is significant difficulty incorporating BDE of the weakest bond into the scoring function for a generative model. First, the cost associated with accurate predictions for all BDEs in a given molecule is prohibitively expensive (i.e., it scales approximately as 3·*n* times the cost of orbital energy calculations where *n* is the number of bonds per molecule). Second, the BDE for the weakest bond of an entire molecule is extremely sensitive to the subtle changes in the molecule’s chemical structure and conformation. A machine learning approach for prediction is nearly impossible, and further, we note this is a different problem than predicting a single BDE of a particular bond given surrounding chemistry (St. John et al., 2020).

We introduced a post-generative screening stage to refine the machine-generated and model-augmented designs by the weakest BDEs of compounds. This works particularly well with hole transport material design since the training set for the prior network is based on a partially conjugated organic space that is not expected to show the instability typical in other design spaces such as high-energy materials.

For all design examples shown in this work, BDEs of the weakest bond at the ground state (S_0_) and the triplet excited state (T_1_) were computed by quantum chemical simulation using LACV3P** basis set and B3LYP functional. Given the environment in which the hole transport layers are often deployed, BDEs of the cationic (*i.e.*, positively charged) and the anionic states (*i.e*., negatively charged) were also computed. For each molecule, the BDE of the weakest bond was determined by calculating BDEs computed for the bonds that can dissociate without involving hydrogen abstraction processes. Each BDE is computed as the energy difference between the homolytic dissociation of a single, acyclic bond from the input molecular compound. For the excited state BDEs, the energy before the bond dissociation was computed at the designated excited state (*i.e.*, S_1_ or T_1_), representing the initial state for the bond dissociation process. For the charged state BDEs, one electron was added to (for anionic BDE) or removed from (for cationic BDE) the molecule before and after the bond dissociation. Since one can place the extra charge on either side of the dissociated species, each bond results in two independent BDEs for each charged state. This algorithm was automated to be performed over all input structures for all initial states—*i.e.*, neutral, excited, cationic, and anionic. Once the BDEs were computed, they were sorted to identify each state’s lowest BDE per compound. The BDE analysis based on the weakest link of each compound ensures the chemical design created or inspired by the generative model meets the minimum requirements of compound stability under the normal operation of the device.

## *De Novo* Design Strategy and Outlook

### Design Augmented by the Generative Model

The new chemistry drawn from the generative model provides a methodology for autonomous materials design. While the raw chemistry generated as SMILES strings from the generative model shows a list of interesting molecular structures paired with desired properties, much greater insights can be extracted from the individual design suggestions.

First, the generative model methodology demonstrates a viable approach to autonomous material design by delivering viable hole transport structures. While the diversity of structures in this first stage does not take explicit synthetic difficulty into account, it does, however, and minimize the bias a practicing chemist brings to molecular design. Second, one can more efficiently examine a structural space and ensure it has been thoroughly investigated versus the traditional empirical approaches. Third, the model accurately reflects existing known design rules. For example, successful HTL molecules in the design space minimize lone-pair electrons on heteroatoms that are not sterically hindered (*e.g.,* protected) as they may be susceptible to known degradation paths such as photochemical or cationic-nucleophilic reactions, see Figure 8 (So and Kondakov, 2010; Schmidbauer et al., 2013).

While these unwanted characteristics can be filtered out in generating new chemistry, this will result in the loss of a significant number of new candidates generated by the model and thus, interfere with the value we get from examining a diverse set. A recommended strategy in the putative workflow is to investigate the representative groups of compounds that share frequently appearing substructure patterns, then apply minor corrections in their design by manual inspection.

**FIGURE 9:**
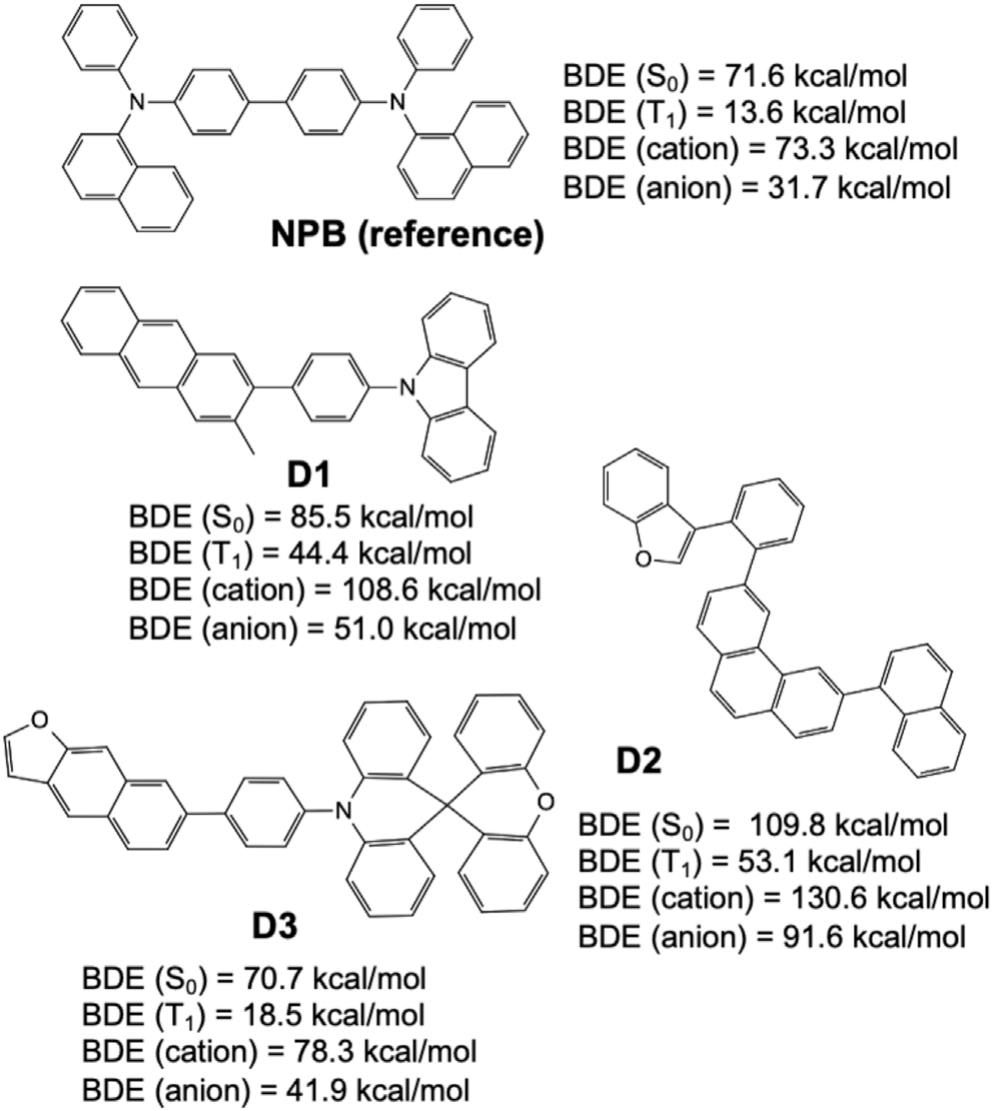
Bond dissociation energies for the ground state (S_0_), triplet-excited state (T_1_), cationic state, and anionic state, predicted by quantum chemical calculations over three example hole transport material designs derived from the generative model (**D1**, **D2**, and **D3**), and compared to a known hole transport material, NPB (shown on top).

Figure 9 shows select candidates (**D1**, **D2**, and **D3**) derived from structures frequently appearing in the validation set and having high MPO scores while incorporating fewer less-desirable fluorination and alkylation substituents. For example, it has been reported that fluorine substituents on aromatic and heterocyclic ring systems are prone to a variety of chemistries (*e.g.*, homolytic and heterolytic fragmentation or even substitution nucleophilic aromatic (SNAr) reactions with nucleophiles such as pyridyl nitrogen (March 1985; Shi et al., 2015). Further, alkyl substituents that are para- or ortho-to an *sp3* hybridized nitrogen (triarylamine) are suspected to be sites for chemical degradation through free-radical formation, but this has yet to be proven experimentally. Again, the refined set of structures were (re)validated with relatively high MPO scores (MPO_**D1**_ = 0.78, MPO_**D2**_ = 0.72, and MPO_**D3**_ = 0.95), and then further examined for their chemical stability represented by the dissociation energies of the weakest bond, relative to that of a reference hole transport material, NPB (N,N′-Di (1-naphthyl)-N,N′-diphenyl-(1,1′-biphenyl)-4,4′-diamine, and chemical structure shown in Figure 9), on the different potential energy surfaces reviewed (*e.g.*, ground state, triplet-excited state, cationic, and anionic states), confirm that all structures were valid hole transport material candidates.

Another design rule can be extracted from the generative model by examining the pattern of how the machine draws the new compounds rather than simply accepting computed recommendations. The success of this methodology is evidenced by the similarity analysis summarized in Figure 7; many chemical motifs from the training set for the prior network reappear in the candidates from the generative model.

**FIGURE 10:**
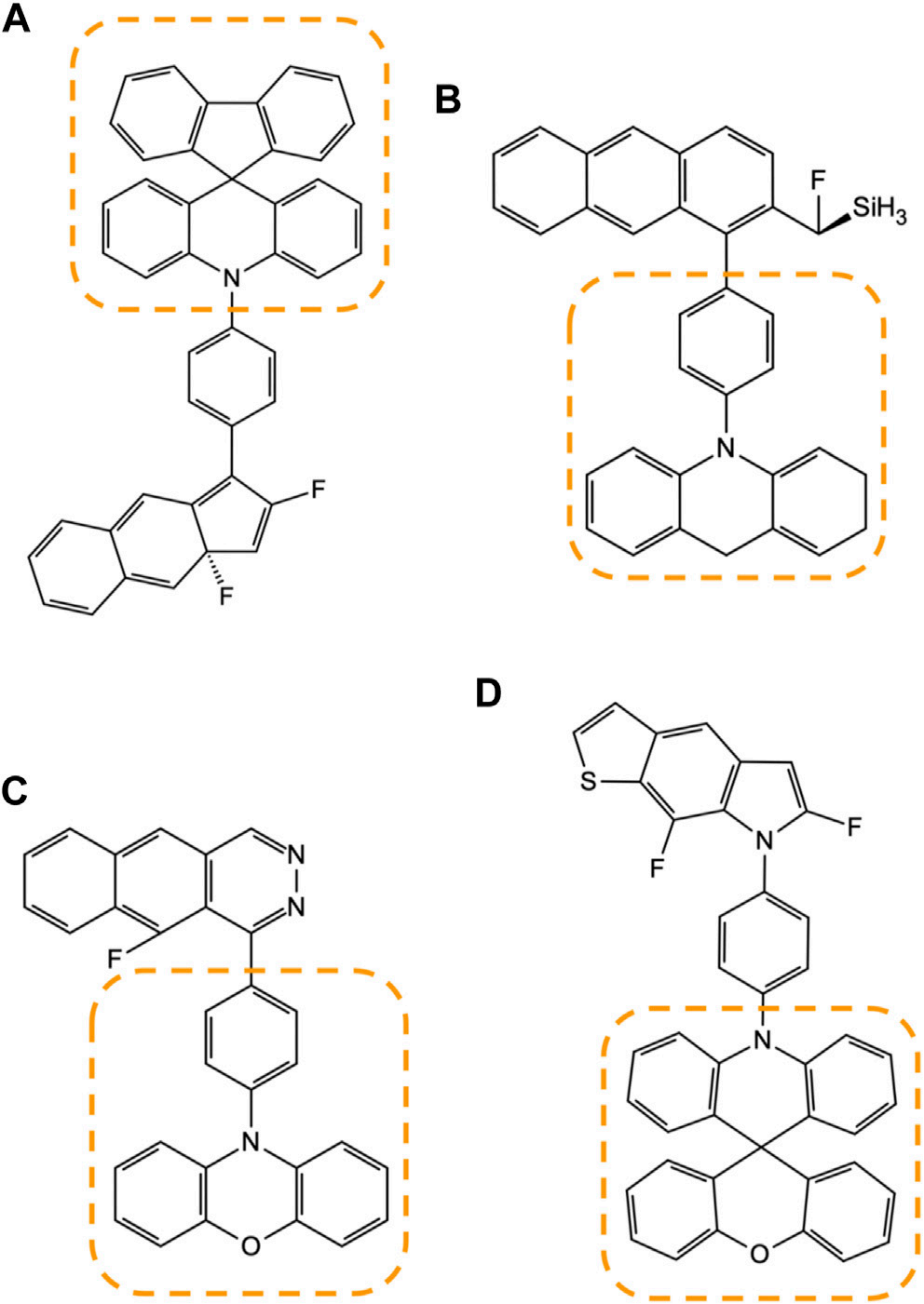
Four examples **(A–D)** of the high-MPO-scoring compounds from the generative model are accompanied by some of the frequently appearing substructure motifs, marked by the area enclosed within the dotted lines.

Figure 10 illustrates how these frequently appearing motifs are identified amongst the candidate compounds generated by the model Figures 10A–D. During the manual inspection of several high-scoring compounds, we found that a particular set of structural patterns appear with higher frequency than others, and such as those marked in the figure with the dotted lines. We identified that these patterns are tied to the design rules that are not as obvious to the human eye and can be used to create another set of more advanced designs that neither human nor machine has considered.

The advantage of this strategy is that unlike blindly accepting the outcome of the generative model, one could get access to a much clearer logic behind the structure-property relationships. It also presents an opportunity for the materials experts to seamlessly compare their ideas and intuition with the machine model results.

Not surprisingly, most of the frequently appearing substructures are among the fragments found in either the core or the R-group chemistry used to build the prior network. This means the design process relies on concentrating the existing chemical design space on what works best rather than digging up an entirely new chemical space every time. This design approach represents an enrichment of the chemical space.

At the same time, the generative model does not systematically inhibit the appearance of a brand-new chemical motif. Figure 10D is an example of the substructure marked by the dotted line, spiro[acridine-9,9′-xanthene] group, which has also appeared in **D3** and had never been introduced as part of the core chemistry used to build the prior network.

Based on the recognized substructure patterns among the high-scoring candidates returned by the generative model, we built a small set following these simple design rules:

(1) Assemble a structure with two or fewer substructures identified from the raw design
(2) Maintain high symmetry for synthetic viability
(3) Use fast QM calculations, including the BDE analysis, and to iterate through ideas

**FIGURE 11:**
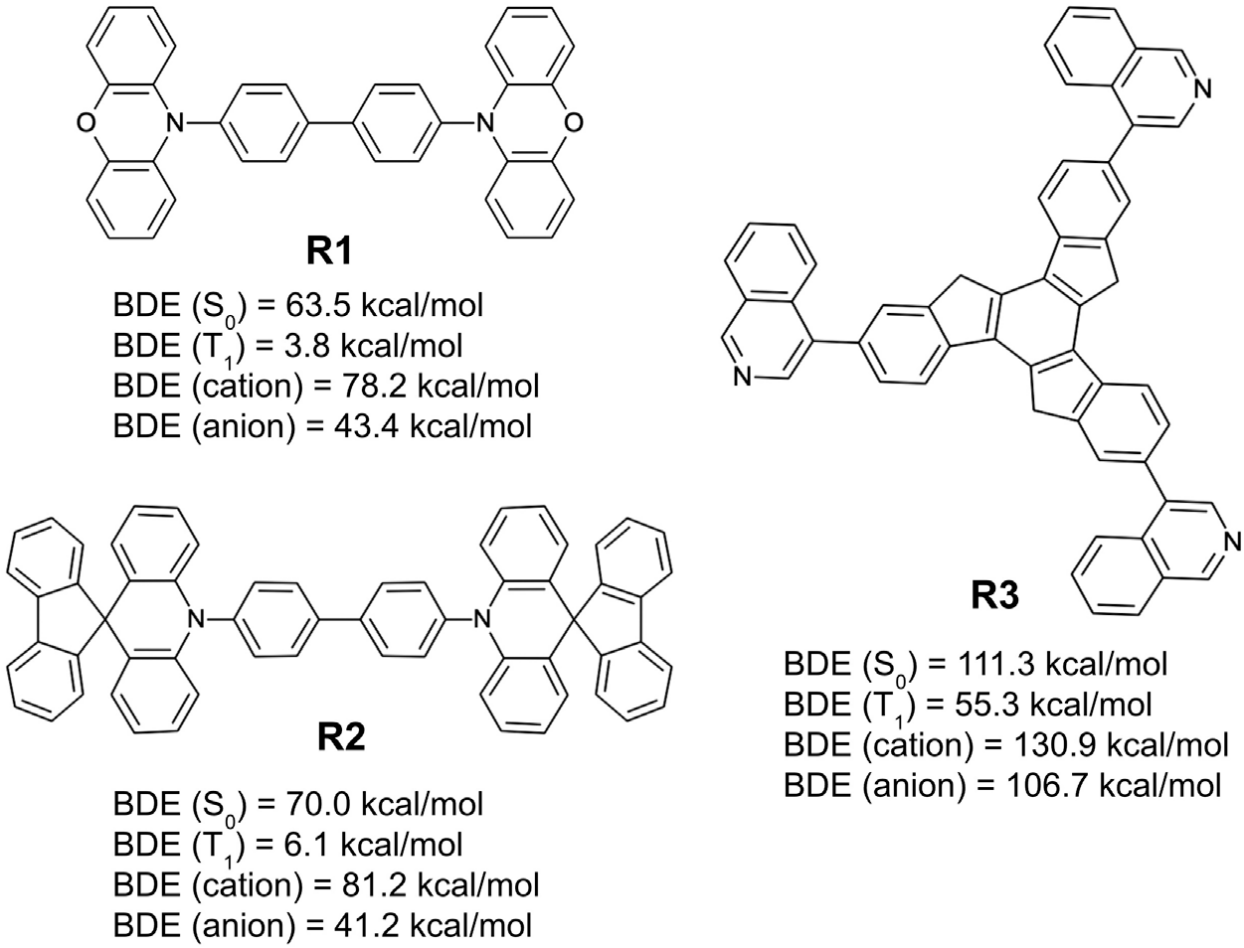
Bond dissociation energies for the ground state (S_0_), triplet-excited state (T_1_), cationic state, and anionic state, predicted by quantum chemical calculations over three example hole transport material designs derived from the generative model **(R1, R2, and R3)** derived from the design principle that takes the patterns observed by the generative model as input while coupling them with user directives.

Figure 11 lists three of the simplest design examples following these rules. Among the examples shown in the figure, two (**R1** and **R2**) are from simple recombination of the frequently appearing chemical substructures from the generative model, 10-phenyl phenoxazine and 10-phenyl 10H-spiro acridine-9,9′-fluorene, respectively. They are based on a primary design principle that combines the same symmetry point at the center (2-fold rotational) and the core chemistry (biphenyl) of NPB. The remaining structure (**R3**) is an extension of a known chemical motif, truxene (Yang et al., 2009; Goubard and Dumur, 2015; Shi et al., 2015), and with an R-group (isoquinoline) that appears in the machine-drawn candidates with high frequency.

The example designs **R1–R3** were assessed for their predicted properties in the same manner as previously done (**D1–D3**), and the MPO scores for all three remain quite high (MPO_**R1**_ = 0.88, MPO_**R2**_ = 0.97, and MPO_**R3**_ = 0.95). The dissociation energy of the weakest bonds for **R1** and **R2** in the S_0_ and T_1_ states are predicted to be equal to or lower than those of NPB. **R3** looks to have significantly better chemical stability, given that the BDE from the weakest bond is expected to be higher than the other two by nearly 50 kcal/mol for all examined states. BDE of the weakest bonds for the cationic and the anionic surfaces are predicted to be higher than that of NPB, confirming a legitimate design example for novel hole transport materials that can withstand both hole and electron currents. MPO scores and the individual material properties (including the results from BDE analysis) of the six example designs are summarized in Table 2 for a quick comparison.

**TABLE 2:** Summary of MPO, individual design properties, and BDE analysis results for the machine-drawn design examples (**D1–D3**) and recombination with frequently appearing structural patterns (**R1–R3**).

Design	MPO	*E*_HOMO_ (eV)	*E*_LUMO_ (eV)	*λ*_*h*_ (eV)	*T*_*g*_ (°C)	Weakest BDE (kcal/mol)			
						*S*_*0*_	*T*_*1*_	*Cation*	*Anion*
**D1**	0.78	−5.77	−2.75	0.12	125	85.5	44.4	108.6	51.0
**D2**	0.72	−5.43	−2.44	0.18	120	109.8	53.1	130.6	91.6
**D3**	0.95	−5.57	−2.61	0.14	168	70.7	18.5	78.3	41.9
**R1**	0.88	−5.36	−2.49	0.08	158	63.5	3.8	78.2	43.4
**R2**	0.97	−5.59	−2.42	0.05	184	70.0	6.1	81.2	41.2
**R3**	0.95	−6.06	−2.60	0.14	164	111.3	55.3	130.9	106.7

While the design rules illustrated in this work demonstrate solid pathways to novel organic electronic materials using unbiased, machine-generated information, and these examples also highlight that the human factor cannot be completely isolated. The discontinuity of the structure-property relationship is often quite evident after minor refinement. Still, it may be easily missed by a non-expert. One example of this issue is exemplified with the design of **R1** and **R2**. By following the same design principles (1–3) illustrated above, there is no apparent pressure to favor either biphenyl groups or a single phenyl group at the center. However, an experienced OLED expert would immediately note that the biphenyl groups at the center are observed in successful hole transport materials (*e.g.,* NPB, CBP, TPD, and dPVBi, etc.). A quick set of quantum chemical calculations supports the expert’s intuition. In this case, the single-phenyl-ring versions of **R1** and **R2** end up with lower bond dissociation energies around the core, especially with near-zero or even negative BDE in the T_1_ state.

This does not mean a single phenyl ring near the center should be eliminated from design directives. As seen for **D1** and **D2**, the machine found a set of chemical motifs around a single phenyl ring that delivers adequate stability while satisfying critical requirements for hole transport materials. This is an example where the machine can find subtle yet often not-readily-interpretable changes outside the desirable core chemistry, which effectively drive the property space further into the more desired target space, and as shown in Figure 8. In both cases, the clear benefit of the design augmented by the generative model is that one could form a high-throughput process whereby a machine dispenses unbiased design ideas while human experts constantly filter and refine the list, nudging it in the direction of viable design rules. In other words, the machine becomes an expert tool to accelerate the creation of new design ideas while diversifying the design space in an unbiased manner. But, it is not a replacement for human experts. Instead, it is a tool that augments experts and thus accelerates exploration of a target material space.

### Chemistry by Machine: The Next Step

While this work demonstrates the value of using a generative model approach for materials design, many challenges remain. One of them is to account for the subtle balance between chemical diversity and synthesizability in the autonomous chemical design process. In this work, R-group enumeration from known core chemistry and symmetric attachment points was utilized as a simple measure to circumvent the synthesizability issue without explicitly involving additional scoring functions for synthetic viability. This approach produced new chemistry resembling the available design space and identified novel hole transport materials while maintaining synthetic viability. A next step to expand this work would involve constructing a neural network for the generative model with an explicit synthesizability score integrated into the MPO score. The main hurdle for such an approach is the availability of tools that offer sufficient accuracy over the entire molecular space (Boda et al., 2007; Ertl and Schuffenhauer, 2009; Emami et al., 2015; Schneider et al., 2016; Coley et al., 2018; Konze et al., 2019). Another method uses the transfer learning approach (Merk et al., 2018), which partially retrains the prior networks for a smaller set of known synthesizable structures. This has been shown to enable the agent networks to be biased toward generating more synthesizable structures for an RNN-based generative model of drug discovery (Segler et al., 2018). Such an approach could potentially drive the design process to start with a much more diverse chemical space, then follow up by fine-tuning the design selections with a smaller set of known hole transport materials. The first step towards this is to survey algorithms to produce synthetic accessibility (SA) generally accepted in the community, generate the SA scores for the training set plus the generated set of compounds in this work, and perform a comparative analysis to assess the validity as well as to enhance the MPO scoring scheme towards the synthesizability. We believe this could be an immediate future work, explicitly considering a more extensive set of operational design rules for OLED materials lifetime.

Aside from refining the algorithms to generate new and improved materials chemistry from the generative model, human intuition and how its construction is integrated into the design process plays a critical role in the goal-directed materials discovery process. The hole transport materials example illustrates the importance of expert input when setting up the desired property space for the model and defining the final design rules that are transferable and interpretable. Ultimately, this is not a single stream design process that starts with fixed neural networks and ends with a few refinements. It is designed to work as an iterative process where the neural network and the scoring scheme get updated with new information and insights obtained recursively from the design ideas and the generative model.

## Conclusion

A goal-directed generative model powered by recurrent neural networks and reinforcement learning algorithms is presented as a new materials discovery strategy in organic electronic applications. High-throughput quantum mechanical calculations and automated machine learning algorithms were combined with the RNN-based generative model to produce novel design ideas for molecular materials based on the desired set of target properties and design rules. Demonstration of the framework with a hole transport material example confirms its value as a design tool that accelerates the materials discovery process based on an unbiased sampling of chemical space while optimizing the properties of the materials.

Retaining similarity to the existing hole transport material design space, the generative model successfully created a new set of design ideas while optimizing for target properties, and exemplified by a multiparameter optimization (MPO) score of 92% compared to related structures in the training set chemistry. Despite using a single MPO score to define the goal in the property space, improvements of all individual properties (*e.g.*, orbital energies, hole reorganization energy, and glass transition temperature) were observed across the design suggestions. Utilizing a few expert design principles, one can construct machine-generated design ideas that are used as valuable ingredients for a set of novel design directives that are set to find the balance between chemical diversity and synthetic viability.

We note that the materials design process presented in this work by the goal-directed generative model is not confined to the organic electronics space but introduces a new data-driven design framework for molecular materials. We believe the introduction of the framework will significantly reduce the development cost for new molecular materials, enabling rapid exploration, and validation of a chemical design space.

## Data Availability

The original contributions presented in the study are included in the article/Supplementary Material, further inquiries can be directed to the corresponding authors.

## References

[B1] Abd El-Karim S. S. Anwar M. M. Syam Y. M. Nael M. A. Ali H. F. Motaleb M. A. (2018). Rational Design and Synthesis of New Tetralin-Sulfonamide Derivatives as Potent Anti-diabetics and DPP-4 Inhibitors: 2D & 3D QSAR, *In Vivo* Radiolabeling and Bio Distribution Studies. Bioorg. Chem. 81, 481–493. 10.1016/j.bioorg.2018.09.021 30243239

[B2] Afzal M. A. F. Browning A. R. Goldberg A. Halls M. D. Gavartin J. L. Morisato T. (2021). High-Throughput Molecular Dynamics Simulations and Validation of Thermophysical Properties of Polymers for Various Applications. ACS Appl. Polym. Mater. 3 (2), 620–630. 10.1021/acsapm.0c00524

[B3] Alperstein Z. Cherkasov A. Rolfe J. T. (2019). All Smiles Variational Autoencoder. arXiv.

[B4] An Y. Sherman W. Dixon S. L. (2012). Hole Filling and Library Optimization: Application to Commercially Available Fragment Libraries. Bioorg. Med. Chem. 20, 5379–5387. 10.1016/j.bmc.2012.03.037 22503740

[B5] An Y. Sherman W. Dixon S. L. (2013). Kernel-Based Partial Least Squares: Application to Fingerprint-Based QSAR with Model Visualization. J. Chem. Inf. Model. 53 (9), 2312–2321. 10.1021/ci400250c 23901898

[B6] Bartók A. P. De S. Poelking C. Bernstein N. Kermode J. R. Csányi G. (2017). Machine Learning Unifies the Modelling of Materials and Molecules. Sci. Adv. 3, e1701816. 10.1126/sciadv.1701816 29242828 PMC5729016

[B7] Boda K. Seidel T. Gasteiger J. (2007). Structure and Reaction Based Evaluation of Synthetic Accessibility. J. Comput. Aided Mol. Des. 21, 311–325. 10.1007/s10822-006-9099-2 17294248

[B8] Brown N. Fiscato M. Segler M. H. S. Vaucher A. C. (2019). GuacaMol: Benchmarking Models for De Novo Molecular Design. J. Chem. Inf. Model. 59, 1096–1108. 10.1021/acs.jcim.8b00839 30887799

[B9] Buckley A. (2013). Organic Light-Emitting Diodes (OLEDs). Philadelphia: Woodhead Publishing Lmtd.

[B10] Butler K. T. Davies D. W. Cartwright H. Isayev O. Walsh A. (2018). Machine Learning for Molecular and Materials Science. Nature 559, 547–555. 10.1038/s41586-018-0337-2 30046072

[B11] Cheng J. Y.-J. Frangos C. Groysberg B. (2021). Harvard Business Review.

[B12] Coley C. W. Rogers L. Green W. H. Jensen K. F. (2018). SCScore: Synthetic Complexity Learned from a Reaction Corpus. J. Chem. Inf. Model. 58 (2), 252–261. 10.1021/acs.jcim.7b00622 29309147

[B13] de Oliveira M. T. Katekawa E. (2018). On the Virtues of Automated Quantitative Structure-Activity Relationship: the New Kid on the Block. Future Med. Chem. 10, 335–342. 10.4155/fmc-2017-0170 29393678

[B14] Dixon S. L. Duan J. Smith E. Von Bargen C. D. Sherman W. Repasky M. P. (2016). AutoQSAR: an Automated Machine Learning Tool for Best-Practice Quantitative Structure-Activity Relationship Modeling. Future Med. Chem. 8, 1825–1839. 10.4155/fmc-2016-0093 27643715

[B15] Emami F. S. Vahid A. Wylie E. K. Szymkuć S. Dittwald P. Molga K. (2015). A Priori Estimation of Organic Reaction Yields. Angew. Chem. Int. Ed. 54 (37), 10797–10801. 10.1002/anie.201503890 26215084

[B16] Ertl P. Schuffenhauer A. (2009). Estimation of Synthetic Accessibility Score of Drug-like Molecules Based on Molecular Complexity and Fragment Contributions. J. Cheminform 1, 8. 10.1186/1758-2946-1-8 20298526 PMC3225829

[B17] Evans D. R. Kwak H. S. Giesen D. J. Goldberg A. Halls M. D. Oh-e M. (2016). Estimation of Charge Carrier Mobility in Amorphous Organic Materials Using Percolation Corrected Random-Walk Model. Org. Electro. 29, 50–56. 10.1016/j.orgel.2015.11.021

[B18] Fujikawa H. Ishii M. Tokito S. Taga Y. (2000). Organic Light-Emitting Diodes Using Triphenylamine Based Hole Transporting Materials. MRS Symp. Proc. 621, Q3.4.1. 10.1557/proc-621-q3.4.1

[B19] Gao Z. Q. Li Z. H. Xia P. F. Wong M. S. Cheah K. W. Chen C. H. (2007). Efficient Deep-Blue Organic Light-Emitting Diodes: Arylamine-Substituted Oligofluorenes. Adv. Funct. Mater. 17, 3194–3199. 10.1002/adfm.200700238

[B20] Ghanakota P. Bos P. H. Konze K. D. Staker J. Marques G. Marshall K. (2020). Combining Cloud-Based Free-Energy Calculations, Synthetically Aware Enumerations, and Goal-Directed Generative Machine Learning for Rapid Large-Scale Chemical Exploration and Optimization. J. Chem. Inf. Model. 60 (9), 4311–4325. 10.1021/acs.jcim.0c00120 32484669

[B21] Ghiringhelli L. M. Carbogno C. Levchenko S. Mohamed F. Huhs G. Lüders M. (2017). Towards Efficient Data Exchange and Sharing for Big-Data Driven Materials Science: Metadata and Data Formats. Npj Comput. Mater. 3, 46. 10.1038/s41524-017-0048-5

[B22] Gómez-Bombarelli R. Aguilera-Iparraguirre J. Hirzel T. D. Duvenaud D. Maclaurin D. Blood-Forsythe M. A. (2016). Design of Efficient Molecular Organic Light-Emitting Diodes by a High-Throughput Virtual Screening and Experimental Approach. Nat. Mater 15, 1120–1127. 10.1038/nmat4717 27500805

[B23] Goubard F. Dumur F. (2015). Truxene: a Promising Scaffold for Future Materials. RSC Adv. 5, 3521–3551. 10.1039/c4ra11559g

[B24] Halls M. D. Giesen D. J. Hughes T. F. Goldberg A. Cao Y. Kwak H. S. (2016). Accelerated Discovery of OLED Materials Through Atomic-Scale Simulation. Proc. SPIE, 9941. 10.1117/12.2237940

[B25] Halls M. D. Yoshidome D. Mustard T. J. Goldberg A. Kwak H. S. Gavartin J. L. (2015). Atomic-scale Simulation for the Analysis, Optimization and Accelerated Development of Organic Optoelectronic Materials. J. Imaging Soc. Jpn. 54, 561–569. 10.11370/isj.54.561

[B26] Himanen L. Geurts A. Foster A. S. Rinke P. (2019). Data‐Driven Materials Science: Status, Challenges, and Perspectives. Adv. Sci. 6, 1900808. 10.1002/advs.201900808 PMC6839624 31728276

[B27] Iwaloye O. Elekofehinti O. O. Oluwarotimi E. A. Kikiowo B. i. Fadipe T. M. (2020). Insight into Glycogen Synthase Kinase-3β Inhibitory Activity of Phyto-Constituents from Melissa Officinalis: In Silico Studies. Silico Pharmacol. 8, 2. 10.1007/s40203-020-00054-x PMC7487069 32968615

[B28] Jennings P. C. Lysgaard S. Hummelshøj J. S. Vegge T. Bligaard T. (2019). Genetic Algorithms for Computational Materials Discovery Accelerated by Machine Learning. Npj Comput. Mater. 5, 46. 10.1038/s41524-019-0181-4

[B29] Jewell J. T. Khazaie V. R. Mohsenzadeh Y. (2021). OLED: One-Class Learned Encoder-Decoder Network with Adversarial Context Masking for Novelty Detection. arXiv.

[B30] Jhulki S. Moorthy J. N. (2018). Small Molecular Hole-Transporting Materials (HTMs) in Organic Light-Emitting Diodes (OLEDs): Structural Diversity and Classification. J. Mater. Chem. C. 6, 8280–8325. 10.1039/c8tc01300d

[B31] Kimura M. Kuwano S. Sawaki Y. Fujikawa H. Noda K. Taga Y. (2005). New 9-fluorene-type Trispirocyclic Compounds for Thermally Stable Hole Transport Materials in OLEDs. J. Mater. Chem. 15, 2393–2398. 10.1039/b502268a

[B32] Kondakova M. E. Pawlik T. D. Young R. H. Giesen D. J. Kondakov D. Y. Brown C. T. (2008). High-efficiency, Low-Voltage Phosphorescent Organic Light-Emitting Diode Devices with Mixed Host. J. Appl. Phys. 104, 094501. 10.1063/1.3000046

[B33] Konze K. D. Bos P. H. Dahlgren M. K. Leswing K. Tubert-Brohman I. Bortolato A. (2019). Reaction-Based Enumeration, Active Learning, and Free Energy Calculations to Rapidly Explore Synthetically Tractable Chemical Space and Optimize Potency of Cyclin-dependent Kinase 2 Inhibitors. J. Chem. Inf. Model. 59 (9), 3782–3793. 10.1021/acs.jcim.9b00367 31404495

[B34] Lee H. Park J. H. Yang K. J. Hwang S. J. Braveenth R. Ha T. H. (2021). CN-substituted Ortho-Terphenyl Core Based High Triplet Energy Bipolar Host Materials for Stable and Efficient Blue TADF Devices. J. Mater. Chem. C. 9, 7426–7435. 10.1039/d1tc01119g

[B35] Lim J. Ryu S. Kim J. W. Kim W. Y. (2018). Molecular Generative Model Based on Conditional Variational Autoencoder for De Novo Molecular Design. J. Cheminform. 10, 31. 10.1186/s13321-018-0286-7 29995272 PMC6041224

[B36] Liu Y. Zhao T. Ju W. Shi S. (2017). Materials Discovery and Design Using Machine Learning. J. Materiomics 3, 159–177. 10.1016/j.jmat.2017.08.002

[B37] LiveDesign (2021). LiveDesign Release 8.11. New York, NY: Schrodinger, LLC.

[B38] March J. (1985). Advanced Organic Chemistry. 3rd Ed. New York: John Wiley & Sons.

[B39] Marques G. Leswing K. Robertson T. Giesen D. Halls M. D. Goldberg A. (2021). De Novo Design of Molecules with Low Hole Reorganization Energy Based on a Quarter-Million Molecule DFT Screen. J. Phys. Chem. A. 125, 7331–7343. 10.1021/acs.jpca.1c04587 34342466

[B40] Matsuzawa N. N. Arai H. Sasago M. Fujii E. Goldberg A. Mustard T. J. (2020). Massive Theoretical Screen of Hole Conducting Organic Materials in the Heteroacene Family by Using a Cloud-Computing Environment. J. Phys. Chem. A. 124 (10), 1981–1992. 10.1021/acs.jpca.9b10998 32069044

[B41] Matsuzawa N. N. Arai H. Sasago M. Fujii E. Goldberg A. Mustard T. J. (2020). Massive Theoretical Screen of Hole Conducting Organic Materials in the Heteroacene Family by Using a Cloud-Computing Environment. J. Phys. Chem. A. 124, 1981–1992. 10.1021/acs.jpca.9b10998 32069044

[B42] Melnyk A. R. Pai D. M. (1990). Photoconductors in Electrophotography. Proc. SPIE, 1253.

[B43] Meredig B. Agrawal A. Kirklin S. Saal J. E. Doak J. W. Thompson A. (2014). Combinatorial Screening for New Materials in Unconstrained Composition Space with Machine Learning. Phys. Rev. B 89, 094104. 10.1103/physrevb.89.094104

[B44] Merk D. Friedrich L. Grisoni F. Schneider G. (2018). De Novo Design of Bioactive Small Molecules by Artificial Intelligence. Mol. Inf. 37, 1700153. 10.1002/minf.201700153 PMC5838524 29319225

[B45] Moosavi S. M. Jablonka K. M. Smit B. (2020). The Role of Machine Learning in the Understanding and Design of Materials. J. Am. Chem. Soc. 142, 20273–20287. 10.1021/jacs.0c09105 PMC7716341 33170678

[B46] Murahari M. Mahajan V. Neeladri S. Kumar M. S. Mayur Y. C. (2019). Ligand Based Design and Synthesis of Pyrazole Based Derivatives as Selective COX-2 Inhibitors. Bioorg. Chem. 86, 583–597. 10.1016/j.bioorg.2019.02.031 30782576

[B47] Murdock S. E. Hughes T. F. Kwak S. H. Goldberg A. Giesen D. J. Cao Y. (2015). Discovery of New Anode SEI Forming Additives Using an In Silico Evolutionary Approach. ECS Trans. 69, 67–74. 10.1149/06901.0067ecst

[B48] Naito K. Miura A. (1993). Molecular Design for Nonpolymeric Organic Dye Glasses with thermal Stability: Relations between Thermodynamic Parameters and Amorphous Properties. J. Phys. Chem. 97, 6240–6248. 10.1021/j100125a025

[B49] Nisbet M. L. Pendleton I. M. Nolis G. M. Griffith K. J. Schrier J. Cabana J. (2020). Machine-Learning-Assisted Synthesis of Polar Racemates. J. Am. Chem. Soc. 142, 7555–7566. 10.1021/jacs.0c01239 32233475

[B50] Olivecrona M. Blaschke T. Engkvist O. Chen H. (2017). Molecular De-novo Design through Deep Reinforcement Learning. J. Cheminform. 9, 48. 10.1186/s13321-017-0235-x 29086083 PMC5583141

[B51] Pilania G. Wang C. Jiang X. Rajasekaran S. Ramprasad R. (2013). Accelerating Materials Property Predictions Using Machine Learning. Sci. Rep. 3, 2810. 10.1038/srep02810 24077117 PMC3786293

[B52] Raccuglia P. Elbert K. C. Adler P. D. F. Falk C. Wenny M. B. Mollo A. (2016). Machine-learning-assisted Materials Discovery Using Failed Experiments. Nature 533, 73–76. 10.1038/nature17439 27147027

[B53] Rogers D. Hahn M. (2010). Extended-Connectivity Fingerprints. J. Chem. Inf. Model. 50, 742–754. 10.1021/ci100050t 20426451

[B54] Schmidbauer S. Hohenleutner A. König B. (2013). Chemical Degradation in Organic Light-Emitting Devices: Mechanisms and Implications for the Design of New Materials. Adv. Mater. 25, 2114–2129. 10.1002/adma.201205022 23450816

[B55] Schneider G. Fechner U. (2005). Computer-based De Novo Design of Drug-like Molecules. Nat. Rev. Drug Discov. 4, 649–663. 10.1038/nrd1799 16056391

[B56] Schneider N. Lowe D. M. Sayle R. A. Tarselli M. A. Landrum G. A. (2016). Big Data from Pharmaceutical Patents: A Computational Analysis of Medicinal Chemists' Bread and Butter. J. Med. Chem. 59 (9), 4385–4402. 10.1021/acs.jmedchem.6b00153 27028220

[B57] Schrödinger (2021). Schrödinger Release 2021-2: Materials Science Suite. New York, NY: Schrödinger, LLC.

[B58] Segler M. H. S. Kogej T. Tyrchan C. Waller M. P. (2018). Generating Focused Molecule Libraries for Drug Discovery with Recurrent Neural Networks. ACS Cent. Sci. 4 (1), 120–131. 10.1021/acscentsci.7b00512 29392184 PMC5785775

[B59] Shahnawaz S. Sudheendran Swayamprabha S. Nagar M. R. Yadav R. A. K. Gull S. Dubey D. K. (2019). Hole-transporting Materials for Organic Light-Emitting Diodes: an Overview. J. Mater. Chem. C. 7, 7144–7158. 10.1039/c9tc01712g

[B60] Shalf J. (2020). The Future of Computing beyond Moore's Law. Phil. Trans. R. Soc. A. 378, 20190061. 10.1098/rsta.2019.0061 31955683

[B61] Shi K. Wang J.-Y. Pei J. (2015). π-Conjugated Aromatics Based on Truxene: Synthesis, Self-Assembly, and Applications. Chem. Rec. 15, 52–72. 10.1002/tcr.201402071 25474741

[B62] Shirota Y. Kageyama H. (2007). Charge Carrier Transporting Molecular Materials and Their Applications in Devices. Chem. Rev. 107, 953–1010. 10.1021/cr050143+ 17428022

[B63] Shirota Y. (2000). Organic Materials for Electronic and Optoelectronic Devices. J. Mater. Chem. 10, 1–25. 10.1039/a908130e

[B64] Shukla D. Welter T. R. Robello D. R. Giesen D. J. Lenhard J. R. Ahearn W. G. (2009). Dioxapyrene-Based Organic Semiconductors for Organic Field Effect Transistors. J. Phys. Chem. C. 113, 14482–14486. 10.1021/jp903472q

[B65] So F. Kondakov D. (2010). Degradation Mechanisms in Small-Molecule and Polymer Organic Light-Emitting Diodes. Adv. Mater. 22, 3762–3777. 10.1002/adma.200902624 20491088

[B66] St. John P. C. Kim Y. Kim S. Paton R. S. Paton R. S. (2020). Prediction of Organic Homolytic Bond Dissociation Enthalpies at Near Chemical Accuracy with Sub-second Computational Cost. Nat. Commun. 11, 2328. 10.1038/s41467-020-16201-z 32393773 PMC7214445

[B67] Truhlar D. G. Cramer C. J. Lewis A. Bumpus J. A. (2004). Molecular Modeling of Environmentally Important Processes: Reduction Potentials. J. Chem. Educ. 81, 596. 10.1021/ed081p596

[B68] Voršilák M. Kolar M. Cmelo I. Svozil D. (2020). SYBA: Bayesian Estimation of Synthetic Accessibility of Organic Compounds. J. Cheminform. 12, 35. 10.1186/s13321-020-00439-2 33431015 PMC7238540

[B69] Wei J. Chu X. Sun X. Y. Xu K. Deng H. X. Chen J. (2019). Machine Learning in Materials Science. InfoMat 1, 338–358. 10.1002/inf2.12028

[B70] Xie Y. Zhang C. Hu X. Zhang C. Kelley S. P. Atwood J. L. (2020). Machine Learning Assisted Synthesis of Metal-Organic Nanocapsules. J. Am. Chem. Soc. 142, 1475–1481. 10.1021/jacs.9b11569 31870151

[B71] Xu J. Chen B. (2005). Prediction of Glass Transition Temperatures of OLED Materials Using Topological Indices. J. Mol. Model. 12, 24–33. 10.1007/s00894-005-0282-5 16133086

[B72] Yang Z. Xu B. He J. Xue L. Guo Q. Xia H. (2009). Solution-processable and thermal-stable Triphenylamine-Based Dendrimers with Truxene Cores as Hole-Transporting Materials for Organic Light-Emitting Devices. Org. Electro. 10, 954–959. 10.1016/j.orgel.2009.04.024

[B73] Yarnell A. Lemonick S. Mullin R. Patel P. (2021). ACS Discovery Report, Q2.

[B74] Yin S. Shuai Z. Wang Y. (2003). A Quantitative Structure−Property Relationship Study of the Glass Transition Temperature of OLED Materials. J. Chem. Inf. Comput. Sci. 43, 970–977. 10.1021/ci034011y 12767156

